# Effects of immunoglobulin Y-loaded amorphous calcium phosphate on dentinal tubules occlusion and antibacterial activity

**DOI:** 10.3389/fbioe.2022.921336

**Published:** 2022-09-28

**Authors:** Yanhong Yan, Yun Guan, Linjuan Luo, Bingqiang Lu, Feng Chen, Beizhan Jiang

**Affiliations:** ^1^ Department of Pediatric Dentistry, Stomatological Hospital and Dental School of Tongji University, Shanghai Engineering Research Center of Tooth Restoration and Regeneration, Shanghai, China; ^2^ Department of Orthopedic, Spinal Pain Research Institute, Shanghai Tenth People’s Hospital, School of Medicine, Tongji University, Shanghai, China

**Keywords:** dentin hypersensitivity, amorphous calcium phosphate, mineralization, antibacterial activity, IgY

## Abstract

**Aim:** This study aimed to evaluate the effects of immunoglobulin Y (IgY)-loaded amorphous calcium phosphate (ACP) (IgY@ACP) on dentinal tubule occlusion and antibacterial activity.

**Methodology:** IgY@ACP was synthesized based on a biomimetic mineralization strategy. The structure was examined by transmission electron microscopy and Fourier transform infrared spectroscopy. The IgY release property was assessed *in vitro*. The cell biocompatibility of IgY@ACP was evaluated by CCK-8. The dentin disks were prepared using healthy human molars, and their dentinal tubules were exposed to EDTA. Subsequently, they were randomly selected and treated with or without IgY@ACP for 7 days. The tubule occlusion morphologies and newly formed layers were observed by scanning electron microscopy (SEM) and x-ray diffraction, respectively. To evaluate the acid resistance and abrasion resistance of IgY@ACP, dentin disks that were treated for 1 day were immersed in acid solution or subjected to a toothbrush. The antibacterial effects against *Streptococcus mutans* (*S. mutans*) were evaluated by colony-forming unit (CFU) counting, adhesion property assessment, and crystal violet and live/dead bacterial staining. Finally, the occlusion effect was evaluated in rat incisors *in vivo*. One-way analysis of variance (ANOVA) was performed for statistical analysis. The level of significance was set at 0.05.

**Results:** IgY@ACP presented an amorphous phase with a nanosize (60–80 nm) and sustained release of protein within 48 h. The CCK-8 results showed that IgY@ACP had good biocompatibility. After treatment with IgY@ACP for 1 day, the majority of dentinal tubules were occluded by a 0.3-μm-thick mineralized layer. Seven days later, all dentinal tubules were occluded by mineralization with a thickness of 1.4 μm and a depth of 16 μm. The newly mineralized layer showed hydroxyapatite-like diffraction peaks. In addition, IgY@ACP had good acid and abrasion resistance. After treatment with IgY@ACP, the CFU counting and adhesion rate of *S. mutans* were significantly reduced, the crystal violet staining was lighter, and the *S. mutans* staining revealed more dead cells. Most importantly, IgY@ACP had a certain occluding property in rat incisors *in vivo*.

**Conclusion:** IgY@ACP can effectively occlude dentinal tubules with acid-resistant stability and has prominent anti-*S. mutans* effects, rendering it a potentially suitable desensitization material in the clinic.

## 1 Introduction

Dentin hypersensitivity (DH) is a common dental symptom that originally results from exposure of dentinal tubules in response to various stimuli such as physical, chemical, mechanical, and other physiological stimuli (Liu et al., 2020). DH often results in severe pain, with a high incidence rate ranging from 4 to 74% (Anthoney et al., 2020). The accepted “hydrodynamic theory” states that external stimuli could cause abnormal movement of fluid within dentinal tubules, stimulating the terminals of pulpal nerve fibers and then causing transient acute pain (Brännström and Aström, 1972). Based on the theory, there are several approaches to DH treatment: 1) the application of desensitizing agents to reduce intra-dental nerve excitability, such as potassium nitrate or potassium chloride (Liu and Hu, 2012). 2) Dental lasers aim to reduce dentin hypersensitivity by coagulating proteins in dentinal tubules, while lasers might destroy the dentin (Ozlem et al., 2018). 3) Physical occlusion of the exposed dentinal tubules, such as glutaraldehyde, oxalate fluoride, calcium sodium phosphosilicate (CSPS; NovaMin, Weybridge, United Kingdom), and casein phosphopeptide-amorphous calcium phosphate-containing crème (CPP-ACP). NovaMin is a bioactive glass material that will release calcium and phosphate ions to form hydroxycarbonate apatite on the dentin surface. Clinical studies showed that toothpastes containing NovaMin are efficacious for DH relief (Hall et al., 2017). CPP-ACP is commonly used for remineralization actin in dentin and enamel, which will lead to the occlusion of the dentinal tubules (Guanipa Ortiz et al., 2019). In addition, due to the defects of enamel and dentin in DH, the exposed dentin is susceptible to dental caries caused by *Streptococcus mutans* (*S. mutans*) (Takahashi and Nyvad, 2016). Therefore, it is highly desirable to develop a biomaterial that can not only effectively alleviate DH by occluding exposed dentinal tubules, but also can simultaneously inhibit *S. mutans* to prevent caries (Kawasaki et al., 2001).

In various desensitization materials based on occluding dentinal tubules, calcium phosphate which has excellent biocompatibility and bioactivity occupies an important position due to chemical properties similar to the inorganic component of human teeth (Prati and Gandolfi, 2015; Berg et al., 2020). The diameter of dentinal tubules is about 4–8 μm, and the pressure of dentinal tubules is outward. The calcium phosphate nanoparticles have facilitation to flow into the dentinal tubules. Then, these nanoparticles can further induce the formation of insoluble mineral deposits on the wall of deep dentinal tubules in the oral environment, to achieve the desired occluding effect on dentinal tubules. There are many strategies to prepare calcium phosphate-based materials. For example, co-precipitation is one of the simple methods to prepare calcium phosphate by using calcium and phosphate salts, which can control the crystal structure to obtain amorphous calcium phosphate (ACP), hydroxyapatite (HA), and other chemical phases (Wang et al., 2017). In these different calcium phosphate materials, ACP, which is usually a precursor of HA, was widely used as a nanocarrier to load and deliver biological macromolecules, such as proteins, peptides, and enzymes (Chen et al., 2011; Qi et al., 2014). It has been reported that ACP was successfully synthesized in simulated body fluid solution (Tas, 2000). As a conventional cell culture medium, Dulbecco’s modified Eagle’s medium (DMEM) has abounded ions of calcium and phosphate, and organic molecules of amino acids, which can simulate the microenvironment of natural biological cells and has indicated a potential for preparing ACP and its complex. Zhou et al. have recently reported a biomimetic mineralization strategy to prepare mineralized ALP nanoparticles (ALP-contained ACP), by using the inter-reaction between ALP and calcium ions from DMEM (Zhou et al., 2022). This biomimetic mineralization strategy has high universality in constructing different protein–ACP complex materials.

Immunoglobulin Y (IgY), the antibody derived from the eggs of hens immunized against anti-*S. mutans*, is normally used for caries treatment (Pereira et al., 2019). As a product of biological immune antibody technology, IgY has the advantages of better biocompatibility and high specificity to cariogenic bacteria. Studies have demonstrated that it is a specific inhibitor against *S. mutans* that prevents it from adhering to the tooth surface by blocking adhesion receptors involved in colonization or aggregation (glucan-binding site) or blocking the activation of glucosyltransferase (GTF) (Krüger et al., 2004; Bachtiar et al., 2016b). In our previous study (Ma et al., 2017), IgY loaded on porous calcium carbonate microspheres was applied to treat DH. But the complex synthesis process and the cytotoxicity of the porous calcium carbonate microspheres limited its clinical conversion, due to its different chemical compositions with the teeth.

In this study, a strategy for the preparation of IgY-loaded ACP (IgY@ACP) has been developed, which combines and maximizes the advantages of IgY and ACP in enabling the dentin surface to combat dentin hypersensitivity and caries. The characterization, biocompatibility, and *in vitro* and *in vivo* effects on dentinal tubule occlusion and antibacterial activity against *S. mutans* of IgY@ACP have been well studied. The results from these experiments have indicated a significant potential of IgY@ACP as a multifunctional material in effectively occlude dentinal tubules and anti-*S. mutans*.

## 2 Materials and methods

**FIGURE 1:**
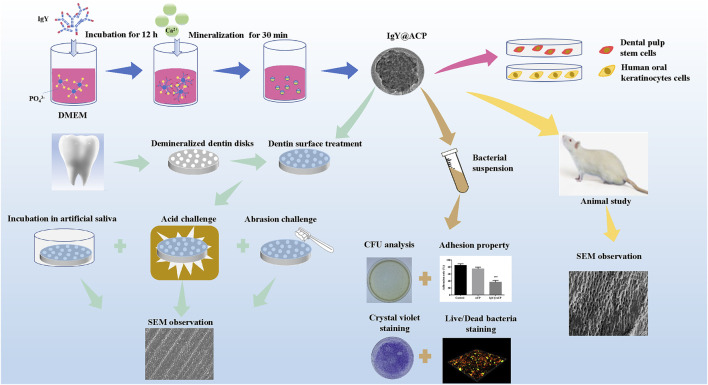
Schematic diagram of the IgY@ACP samples prepared based on the DMEM biomimetic mineralization strategy.

All experiments were approved and performed according to the principles recommended for experimentation with humans and animals established by the Ethics Committee of the Affiliated Stomatology Hospital of Tongji University ([2019]-DW-059). The complete schematic diagram of the IgY@ACP study is shown in Figure 1.

### 2.1 Preparation of IgY@ACP

IgY@ACP was prepared by biomimetic mineralization in Dulbecco’s modified Eagle’s medium (DMEM; HyClone, USA) following a previous study with some modifications (Chen et al., 2017). Briefly, 100 mg of IgY powder (Yasheng Biological Technology, Hangzhou, China) was added to 50 ml of fresh DMEM medium and then incubated in a constant-temperature oscillator (Zhichu, Shanghai, China) for 12 h (37°C, 180 rpm) to reach equilibrium. Afterward, 1 ml of prepared CaCl_2_ (1 mol/L) (Aladdin, Shanghai, China) was added to the previously mentioned solution to induce mineralization of IgY, which was subsequently maintained at 37°C and 5% CO_2_. After incubation for 30 min, the resulting mixture was transferred into centrifugal tubes and centrifuged at a speed of 9,000 rpm/min for 5 min to obtain the precipitates, and the precipitates were thoroughly triple-washed with deionized water to obtain IgY@ACP samples. Based on the preparation procedure mentioned earlier, the corresponding IgY-free calcium phosphate (named ACP) was prepared without the addition of IgY. After centrifugation, some samples were dispersed and kept in absolute ethanol for material characterization and protein determination *in vitro*, and the others were freeze-dried for subsequent studies.

### 2.2 Characterizations of IgY@ACP

The microstructures of ACP and IgY@ACP samples were observed by transmission electron microscopy (TEM, Tecnai G2 F20 S-TWIN, FEI, CA, USA). Briefly, the samples were suspended in ethanol and fully treated in an ultrasonic processor to form dispersions. A drop (50 uL) of dispersion was placed on a copper wire for TEM observation, and selected electron diffraction (SAED) and elemental mapping analysis were performed simultaneously. The size distributions of the samples were obtained by measuring the size of the particles in the TEM micrographs at the same magnification using ImageJ software (National Institutes of Health, Bethesda, MD, USA). Fourier transform infrared (FTIR) was used to analyze the elements in the samples. For this experiment, the KBr pellet technique was used by mixing 1 mg of the powdered sample with 100 mg of spectroscopic-grade KBr in an agate mortar and pressing it into round flakes. The prepared samples were detected using an FTIR spectrometer (FTIR-IS50, Thermo Fisher, Waltham, MA, United States) in the 4,000–400 cm^−1^ wavelength range.

### 2.3 Analysis of IgY release *in vitro*

For assessment of the IgY protein release property *in vitro*, 10 mg of freeze-dried IgY@ACP powder was dissolved in 10 ml of phosphate-buffered saline (PBS; Keygen Biotech, Nanjing, China) (pH = 7.4) and placed in a temperature oscillator with constant shaking (37°C, 180 rpm). At the given time points (5, 10, 20, and 40 min; 1, 2, 3, 6, 12, 18, 24, 30, 42, and 48 h), a certain volume of solution was extracted and centrifuged to collect the supernatant, which was then replaced with an equal volume of fresh PBS solution. The previously mentioned supernatant was incubated with a BCA protein assay kit (Beyotime, Shanghai, China) in a 96-well plate (Corning, USA) for 2 h in the dark to detect protein concentrations at corresponding time points. The OD value of each well at a wavelength of 562 nm was observed with a microplate reader (Biotek, Winooski, Vermont, USA). The protein concentrations of the samples at corresponding time points were calculated according to the standard protein curve equation and the sample volume used, and finally, the cumulative protein release rate curve was generated.

### 2.4 Biocompatibility study

Human oral keratinocytes (HOK) were kindly provided by Professor He Yuan from Tongji University. Human dental pulp stem cells (hDPSCs) were isolated and characterized by flow cytometry as described in our previous study (Xu et al., 2019). HOK and hDPSCs were chosen for cell biocompatibility evaluation using Cell Counting Kit-8 (CCK-8; Beyotime, Shanghai, China). Cells (5×10^3^ cells/well) were seeded in 96-well plates and incubated at 37°C until 80–85% confluence was achieved. Then, DMEM containing 10% fetal bovine serum (FBS; Gibco, CA, USA) with different concentrations (25, 50, 100, and 200 μg/ml) of IgY@ACP and ACP were added into 96-well plates at a volume of 200 μL/well. After incubation for 24 h, all media were removed and washed with PBS twice. Then, 100 μL of DMEM with 10 μL of CCK-8 solution was added to each well (n = 6, each group). The medium without samples served as the control, and the wells without cells were set as the blank control. After 2 h, the absorbance of the supernatant at 450 nm was examined using a microplate reader.

### 2.5 Evaluation of tubule occlusion

The artificial saliva used in our study was prepared following a previous study (Zhang et al., 2018). Then, 0.07 g CaCl_2_, 0.04 g MgCl_2_, 2.23 g KCl, 0.54 g KH_2_PO_4_, and 4.76 g hydroxyethyl piperazine ethanesulfonic acid were dissolved in 80 ml of deionized water, and 0.1 mol/L sodium hydroxide was added to adjust the pH to 7.0. The solution was then transferred to a volumetric flask, and deionized water was added until the solution volume reached 1.00 L. A small amount of thymol was added to prevent fungal growth. The prepared artificial saliva was stored at room temperature for the study. All the aforementioned chemical reagents were purchased from Shanghai Aladdin Biochemical Technology Co., Ltd.

Healthy human molars were collected after informed consent was obtained from the donors and cleaned by ultrasound. Using a slow cutting machine under water cooling, 1 ± 0.1-mm dentin disks were prepared by cutting teeth perpendicular to dentinal tubules, removing the crown enamel, and exposing the middle dentin. The obtained dentin disks were polished with different meshes of silicon carbide (SiC; Goral, USA) sandpaper to make the surface smooth. Then, dentin disks were cleaned with deionized water for 1 min and stored in a 0.1% thymol solution.

#### 2.5.1 The dentinal tubule occlusion of IgY@ACP

Demineralized dentin disks were obtained by immersion in 17% ethylene diamine tetraacetic acid (EDTA; Sigma–Aldrich, MO, USA) for 5 min (João-Souza et al., 2019) to expose the dentinal tubules and were randomly treated with ACP, IgY@ACP, or NovaMin (Sensodyne Repair and Protect, GSK, Canada) (n = 12, each group). In the ACP and IgY@ACP groups, the freeze-dried sample powder and deionized water were fully mixed to make a slurry of samples with a powder/liquid ratio of 10 mg/200 μL. The disposable micro applicator (TPC Advanced Technology, CA, USA) was dipped into the slurry, and the surface of the dentin disks was evenly smeared with the attached samples for 2 min. Then, the treated dentin disks were cleaned with deionized water to remove residual samples, immersed in the prepared artificial saliva, and incubated at 37 °C. Demineralized dentin disks without material treatment immersed in artificial saliva were used as the control group. The artificial saliva was replaced with fresh solution every day. Then, dentin disks that were treated for 1 and 7 days were randomly selected (n = 6, each group), rinsed several times with deionized water, and air-dried at room temperature before examination. The surface and cross-section of all dentin disks were observed using scanning electron microscopy (SEM, S4800, Hitachi, Japan) at 5 kV. Micrographs at magnifications of 1,000× and 5,000× were captured. Subsequently, the crystal orientation and mineral phase of the newly formed layer of dentin disks were analyzed by x-ray diffraction (XRD; Rigaku D/max2500, Japan) and compared with normal and demineralized dentin disks.

#### 2.5.2 Acid resistance and abrasion resistance of IgY@ACP

To test the acid and abrasion resistance of IgY@ACP, dentin disks (n = 6, each group) treated with ACP, IgY@ACP, or NovaMin for 1 day were randomly divided into two subgroups. One subgroup was immersed in a 6% (W/V) citric acid solution (pH = 1.5) for 2 min to test the resistance to strong acid erosion, and the other subgroup was mechanically brushed with a soft-bristled toothbrush (Colgate, NY, USA) at an inclination of approximately 90^o^ with a constant loading for 2 min to test the resistance to daily abrasion. All dentin disks were rinsed several times with deionized water. Then, the surface morphology of the treated dentin disks was observed by SEM at 5 kV.

### 2.6 Antibacterial effects of IgY@ACP

*S. mutans* (ATCC25175) was obtained from the American Type Culture Collection (Manassas, VA, USA). *S. mutans* preserved in cryoprotectant was resuscitated on brain heart infusion (BHI; Haibo, Qingdao, China) agar plates and cultured in a 37 °C anaerobic environment (80% N_2_, 10% CO_2_, and 10% H_2_) for 48 h. A single colony was selected and incubated in a fresh BHI liquid medium for 2–3 generations until the logarithmic growth stage. Then, the bacterial suspension was adjusted to a concentration of approximately 10^5^ colony-forming units (CFUs)/mL prior to use.

#### 2.6.1 CFU analysis

The ACP and IgY@ACP samples were dispersed and diluted to a 10 mg/ml sample suspension in a BHI medium. The bacterial suspension (100 μL) and sample suspension (100 μL) were thoroughly mixed, which obtained a final concentration of 5 mg/ml, and then 200 μL mixture were added to each well of a 96-well plate and incubated in an anaerobic environment at 37°C for 24 h (n = 6, each group). Then, after fully mixing the suspension, 5 μL of each mixture was extracted and diluted ten-fold serially, then 50 μL mixture was added onto BHI agar plates, and the plates were anaerobically cultivated for 48 h. Bacterial suspension and an equivalent volume of BHI medium (without samples) were added to each well as a control group. Then, the CFUs of each plate were photographed by a camera (Canon, Japan) and manually calculated.

#### 2.6.2 Bacterial adhesive study

The adhesion property of *S. mutans* was further investigated. Some original tubes were obtained and divided into the following three groups (n = 6, each group). Group 1: 4.5 ml of ACP (10 mg/ml) suspension with 500 μL of bacterial suspension; Group 2: 4.5 ml of IgY@ACP (10 mg/ml) suspension with 500 μL of bacterial suspension; and Group 3: 4.5 ml of BHI medium with 500 μL of the bacterial suspension. All tubes were placed at a 30^o^ inclination and incubated anaerobically. Take Group 1 as an example. After 24 h of anaerobic culture, the supernatant liquid in the original tube was gently poured into the first new tube, which included nonadhesive bacteria and was named A-1. Subsequently, 5 ml of fresh PBS was added to the original tube, gently washed, shaken, and poured into the second new tube, which included nontightly adhered bacteria and was named B-1. Finally, 5 ml of fresh PBS was added to the original tube, and all the bacteria was scraped off the wall and poured into the third new tube, which included tightly adhered bacteria and was named C-1. Three new tubes were centrifuged, and the supernatants were discarded. Then, after 5 ml of fresh PBS was added and fully mixed, the absorbance of bacteria in the three new tubes was determined at 600 nm by a microplate reader. The adhesion rate of tightly adhered bacteria in Group 1 was obtained by the following calculation: adhesion rate (%) = C-1/(A-1+B-1+C-1) ×100%, where A-1, B-1, and C-1 corresponded to OD 600 values in the three new tubes. Group 2 and Group 3 underwent the same procedure.

#### 2.6.3 Crystal violet staining

The biofilm formation of *S. mutans* was investigated by crystal violet staining. Similarly, the bacterial suspension (500 μL) and sample suspension (500 μL) were thoroughly mixed, and then 1 ml mixture was added to each well of a 24-well plate (Corning, USA) and incubated in an anaerobic environment (n = 6, each group). Bacterial suspension and an equivalent volume of BHI medium (without samples) were added to each well as a control group. After culturing for 24 h, the medium was aspirated, and the remaining bacteria were gently washed with PBS three times. Then, the plate was fixed with 4% (W/V) paraformaldehyde (Sinopharm Chemical, Shanghai, China) for 20 min and washed with PBS three times. At room temperature, 0.1% crystal violet staining solution (Sangon Biotech, Shanghai, China) was added to each well. The staining was applied for 20 min, and the samples were then washed with PBS and photographed by a camera. For quantitative analysis, 400 μL of 95% ethanol (Sinopharm Chemical, Shanghai, China) was added to each well after air drying and shaking for 15 min to dissolve the crystal violet staining solution. The absorbance was measured at 600 nm with a microplate reader. The lighter the dye color was, the less the biofilm that had formed.

#### 2.6.4 Live/dead bacterial staining

To further assess the live and dead status of *S. mutans* in the formed biofilm, live/dead bacterial staining was performed. Similarly, 1 ml of the earlier mentioned mixture was added to the confocal microscopy dishes (Corning, USA) at 37 °C for 24 h in an anaerobic environment (n = 6, each group). Bacterial suspension and an equivalent volume of BHI medium (without samples) were added to each well as a control group. After incubation, the samples were gently rinsed with PBS three times to remove nonadherent bacteria. Subsequently, the *S. mutans* biofilms grown on the dishes were stained using a Live/Dead BacLight Bacterial Viability Kit (40274ES60, Yeasen, Shanghai, China) according to the manufacturer’s instructions. Live bacterial cells were stained with DMAO and, thus, emitted green fluorescence, whereas dead cells were stained with EthD-III and, therefore, emitted red fluorescence. Then, for each dish, a 20-µm-thick 3D image was captured using confocal laser scanning microscopy (CLSM; Carl Zeiss, Oberkochen, Germany) at excitation wavelengths of 530 nm (DMAO) and 620 nm (EthD-Ⅲ). Images were obtained with a ×40 objective, and at least three images were randomly collected from each dish.

### 2.7 Animal study

Six SD rats (6–8 weeks old), weighing 200–250 g, provided 24 teeth (upper and lower incisors) for this study and were randomly divided into the control group and IgY@ACP group. After anesthetization with 2% pentobarbital (Sinopharm Chemical, Shanghai, China) intraperitoneal injection, cavities of 0.3 mm depth were prepared in the buccal and cervical regions of rat incisors using a standard bur (Komet, Germany) on a high-speed handpiece with water cooling. Prior to the treatments, all the cavities were treated with 17% EDTA on small cotton pellets, which were changed every 30 s for 15 min, to remove the smear layer. Each tooth was repeated to smear the IgY@ACP slurry daily for 2 min and then rinsed with PBS. The teeth were treated without IgY@ACP as a control group. After daily treatment for four consecutive days, the animals were anesthetized, and the teeth were carefully extracted to avoid damage to their surface. The tooth surface morphology of the rats was also observed by SEM at 5 kV.

### 2.8 Statistical analysis

All studies were repeated at least three times, and mean values were calculated and expressed with an error bar representing ±SD. SPSS (SPSS 16.0, IBM Corporation, Armonk, NY, USA) was applied for statistical analysis with a one-way analysis of variance (ANOVA). Significant differences were assessed at *p* < 0.05.

## 3 Results

### 3.1 Characterization of IgY@ACP

**FIGURE 2:**
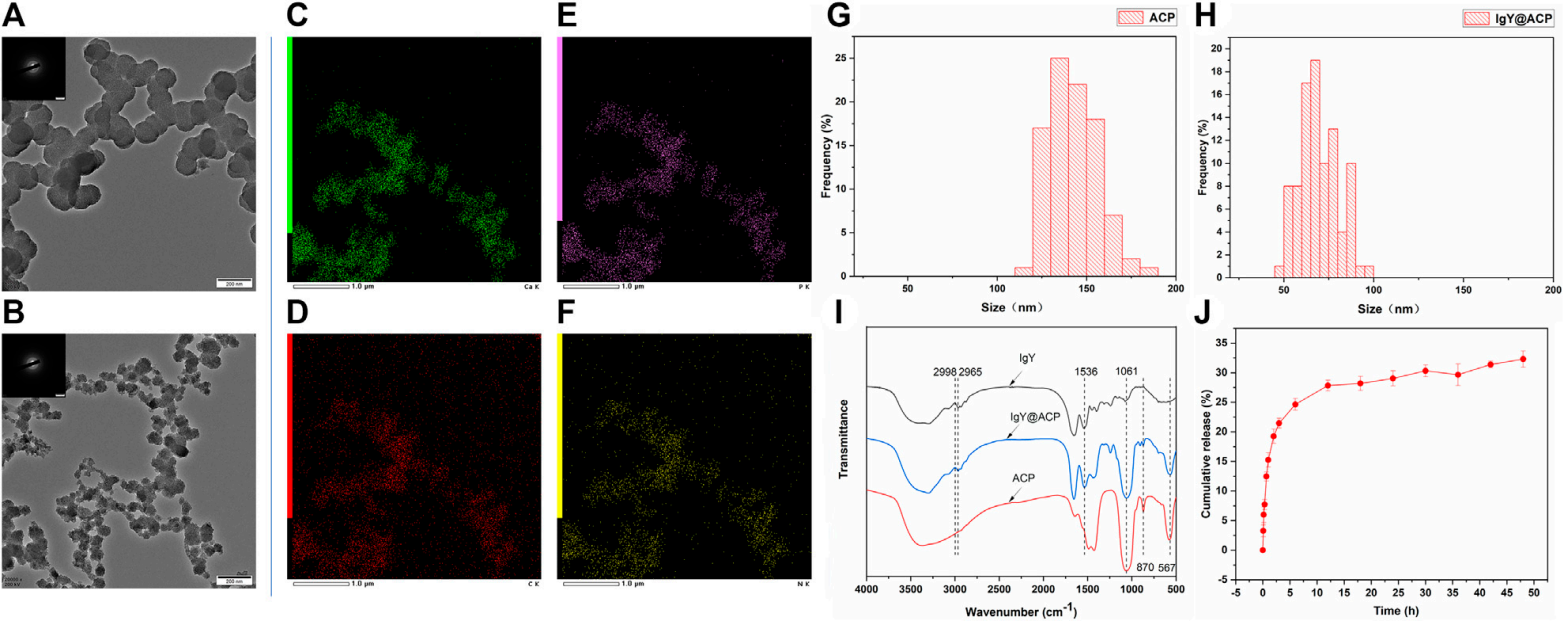
Characterization of ACP and IgY@ACP. TEM images of ACP **(A)** and IgY@ACP **(B)**. Element surface scanning of IgY@ACP **(C–F)**. Size distribution analysis of ACP **(G)** and IgY@ACP **(H)**. FTIR spectra of ACP, IgY, and IgY@ACP **(I)**. The release IgY protein curve of IgY@ACP in PBS **(J)**.

The morphology and particle size of the IgY@ACP composite prepared by the DMEM biomimetic mineralization strategy were observed by TEM. As shown in Figure 2, ACP exhibited the morphology of nanospheres with sizes of approximately 120–160 nm (Figures 2A,G), while IgY@ACP had a similar shape but a much smaller size, 60–80 nm (Figures 2B,H). SAED results indicated that neither sample showed distinct reflections, revealing their amorphous phases. Element mapping analysis revealed that IgY@ACP contained Ca, P, C, and N (Figures 2C–F). In the FTIR spectra of IgY@ACP, the bands at 2,998, 2,965, and 1,556 cm^−1^ could be assigned to IgY molecules, and those at 567, 870, and 1,061 cm^−1^ corresponded to calcium phosphate (Figure 2I).

### 3.2 IgY release *in vitro*

The IgY protein release profiles of IgY@ACP in PBS are shown in Figure 2J. Within the first 2 h, approximately 19.3% of the loaded protein was released into the PBS medium. Then, the IgY protein continued to be released at a gradually slower rate, and the cumulative release rate reached 29.0% at 24 h and 32.3% at 48 h.

### 3.3 Biocompatibility study

**FIGURE 3:**
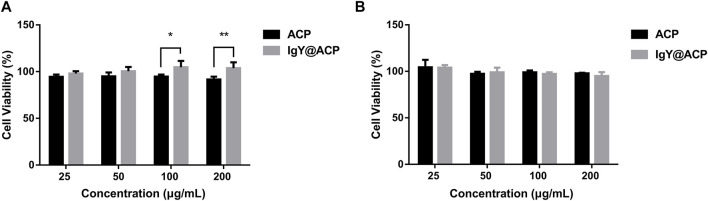
The cell viability of HOK **(A)** and hDPSCs **(B)** cocultured with ACP and IgY@ACP at different concentrations (25, 50, 100, and 200 μg/ml) for 24 h, indicating that the samples had better biocompatibility. Statistical significance was set at *p < 0.05, **p < 0.01, and ***p < 0.001.

The biocompatibility of IgY@ACP and ACP samples was investigated by coculture with HOK cells and hDPSCs and is shown in Figure 3. For HOK cells, the cell viability increased with increasing sample concentration (Figure 3A). Interestingly, the cell viability of HOK cells was much higher in the IgY@ACP group, ranging from 100 to 200 μg/ml (*p* < 0.05). For hDPSCs, the cell viability was above 90% at all concentrations in both samples, with no significant difference (*p* > 0.05) (Figure 3B).

### 3.4 Evaluation of tubule occlusion

**FIGURE 4:**
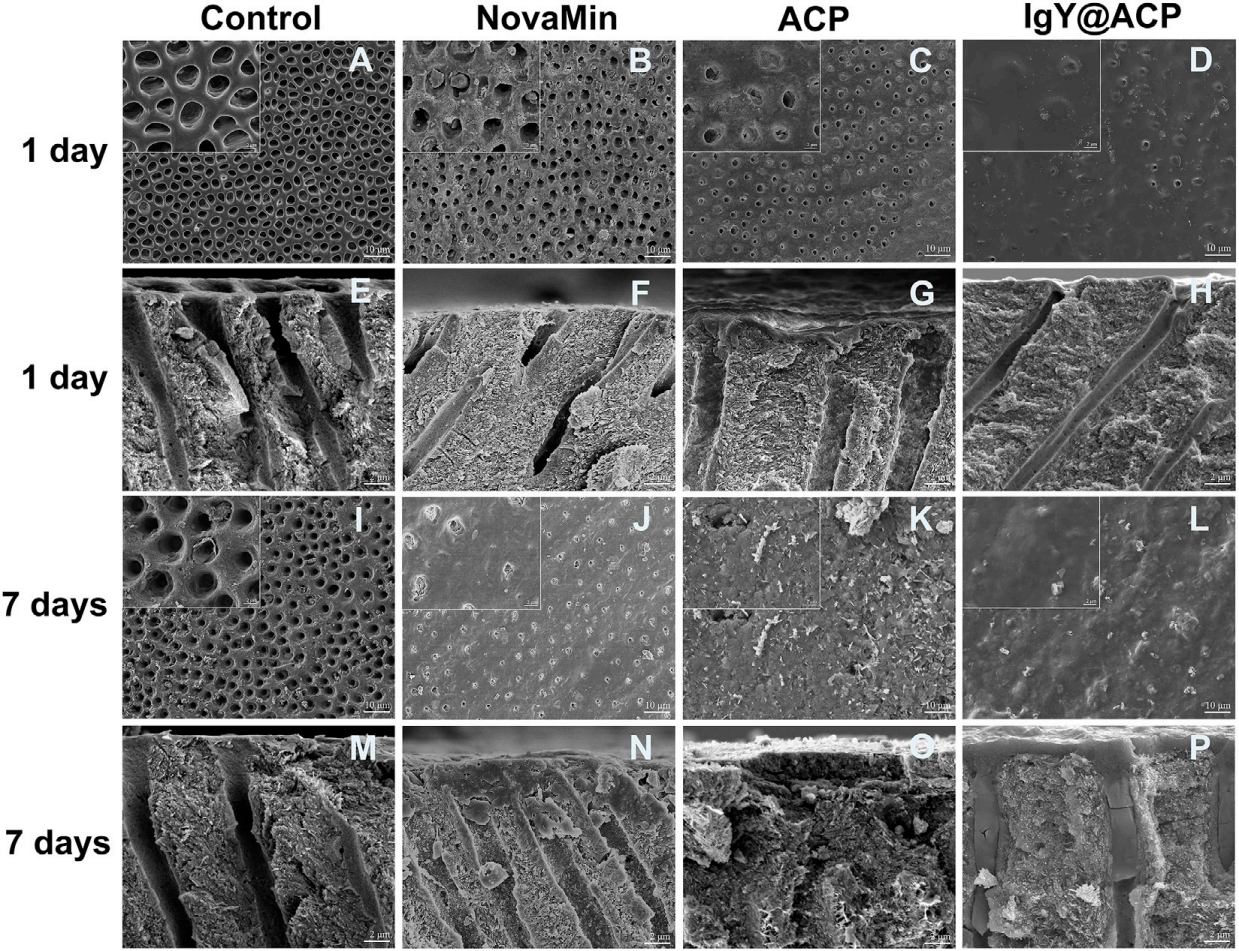
SEM micrographs of the surface and cross-section of dentin disks after treatment with NovaMin **(B,F)**, ACP **(C,G)**, and IgY@ACP **(D,H)** for 1 day and treatment with NovaMin **(J,N)**, ACP **(K,O)**, and IgY@ACP **(L,P)** for 7 days. Dentin disks without treatment for 1 day **(A,E)** and 7 days **(I,M)** were used as the control group.

The surface and cross-sectional images of dentin disks in all groups are shown in Figure 4. As in the DH mimic model, the dentinal tubules in the control group were completely exposed, and the inner space was vacant due to EDTA demineralization (Figures 4A,E,I and M). To treat DH, ACP, IgY@ACP, or NovaMin were evenly applied to the surface of dentin disks, followed by incubation in artificial saliva. After treatment with the NovaMin for 1 day, only a few dentinal tubules were occluded, and the inner space was still vacant (Figures 4B,F). Also, similar results were found in the ACP group (Figures 4C,G). After treatment with IgY@ACP for 1 day, most of the dentinal tubules were occluded by a layer of materials with an average thickness of 0.3 μm, and the remaining opening of nonfully covered tubules was significantly narrowed due to the partially occluding effect of the materials (Figures 4D,H), which was better than that of NovaMin and ACP group. Seven days later, all dentinal tubules were occluded, and sparse lamellar material existed in the inner space in the ACP group (Figures 4K,O). Similarly, all dentinal tubules were occluded, with their surface presenting a thicker (1.4 μm) dense mineralized granular layer in the IgY@ACP group. The cross-sectional images show that the IgY@ACP precipitates intratubularly with a penetration depth of approximately 16 μm and was closely adhered to the wall (Figures 4L,P). However, some tubules were still open in the NovaMin group, with a few particles inside the tubules (Figures 4J,N).

**FIGURE 5:**
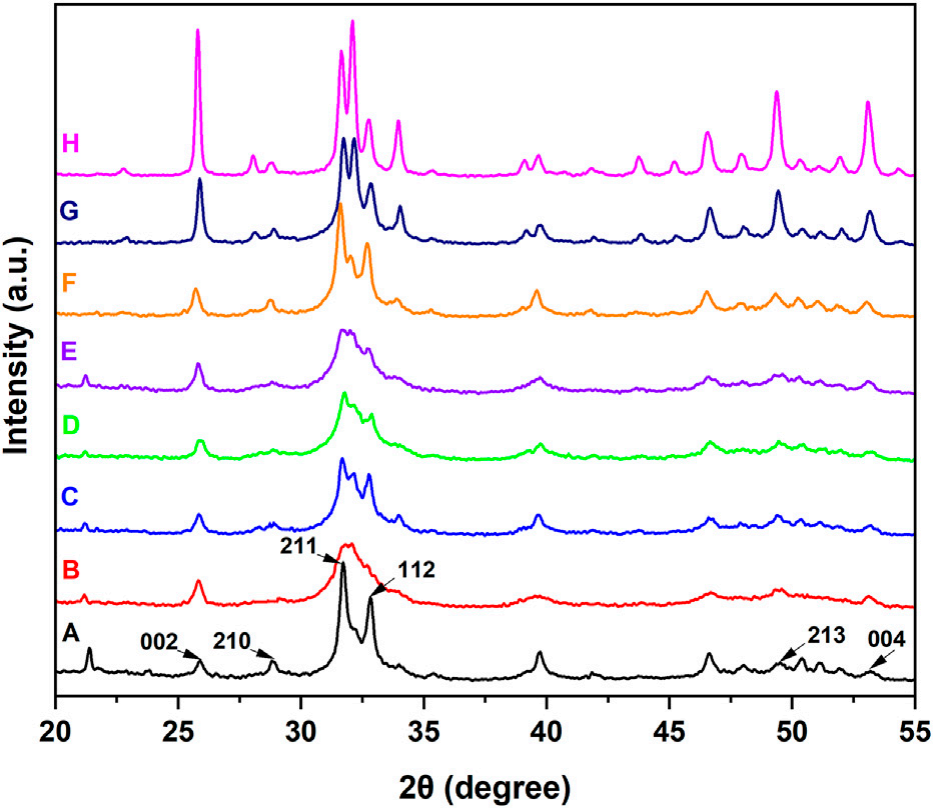
XRD patterns of dentin disks after treatment with NovaMin **(C)**, ACP **(D)**, or IgY@ACP **(E)** for 1 day and treatment with NovaMin **(F)**, ACP **(G)**, or IgY@ACP **(H)** for 7 days. Normal **(A)** and demineralized dentin disks **(B)** were used as the control group.

The types of newly formed crystals on the dentin disks were investigated by XRD. As shown in Figure 5, the diffraction peaks of (002), (210), (211), (112), (213), and (004) at 2θ = 25.9^o^, 28.9^o^, 31.7^o^, 32.8^o^, 49.5^o^, and 53.2^o^, respectively, are characteristic of HAp and appear on the normal dentin disks. In the XRD pattern of demineralized dentin disks, some characteristic peaks, such as those reflected by (210) and (112), were obviously weakened or disappeared. After treatment with ACP or IgY@ACP for 1 day or 7 days, the characteristic diffraction peaks were clearly recovered or even enhanced, which was similar to that of NovaMin.

**FIGURE 6:**
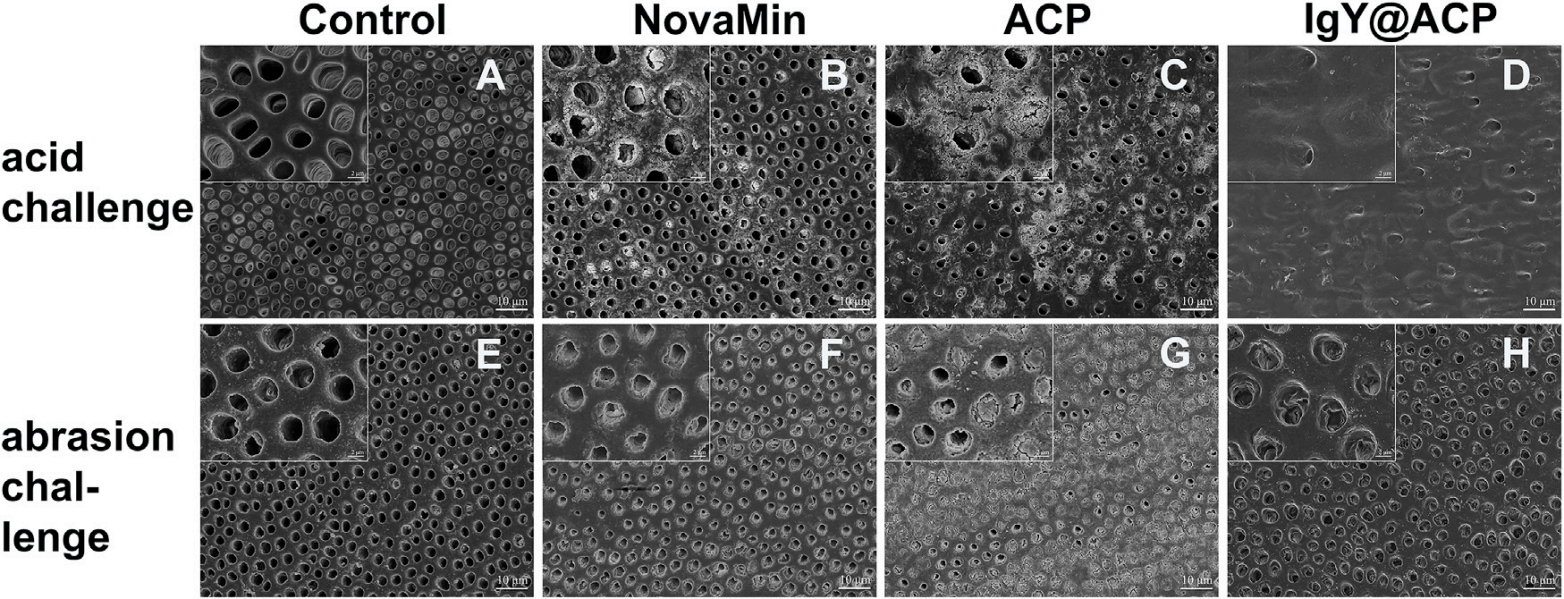
SEM micrographs of dentin disks treated with NovaMin, ACP, or IgY@ACP after 6% critic acid **(B–D)** and abrasion **(F–H)** challenges. Dentin disks without treatment **(A,E)** were used as the control group.

**FIGURE 7:**
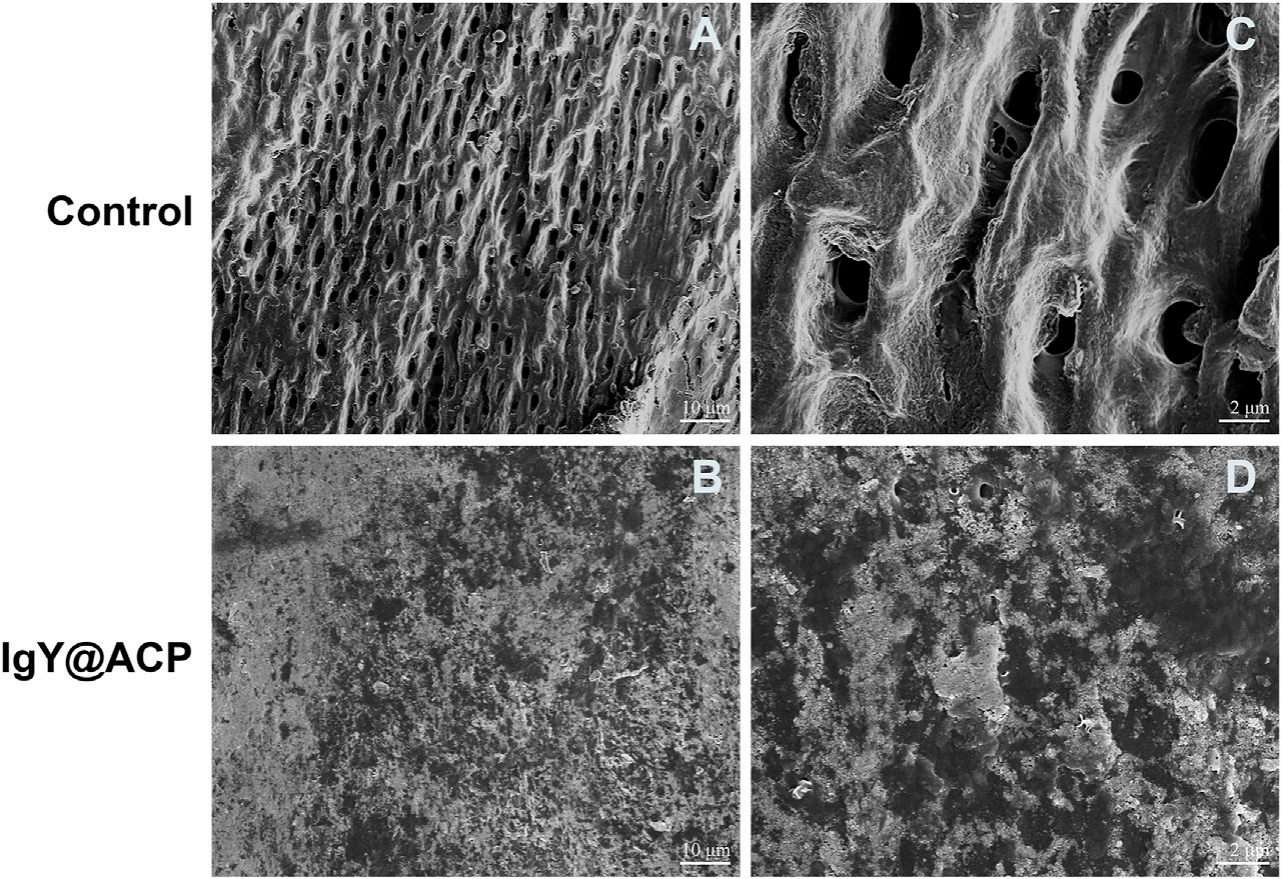
SEM micrographs of the rat dentin surface after treatment with the control **(A)** and IgY@ACP **(B)** for 4 days. **(C)** and **(D)** correspond to the enlarged images of **(A)** and **(B)**, respectively.

The acid- and abrasion-resistances of dentin disks treated with IgY@ACP for 1 day are shown in Figure 6. Compared with the control group, the majority of dentinal tubules remained occluded in the IgY@ACP group after acid treatment, even though a few occluded tubules were damaged and washout traits could be seen on the dentin surface. However, most of the dentinal tubules were exposed in the NovaMin and ACP groups (Figures 6A–D). After the abrasion treatment, compared with the control group, there were still some occluded tubules in the NovaMin and ACP group, and occluding materials still existed in the deep part of the tubules in the IgY@ACP group, although the occluding materials on the dentin surface fell off (Figures 6E–H). In the animal model, the surface images of rat dentin were observed using SEM, as shown in Figure 7. In the control group, most of the dentinal tubules of the rats were exposed to different sizes due to EDTA demineralization (Figures 7A,C). After treatment with IgY@ACP, the dentin surface was covered with mineralized particles, and a few exposed dentin tubules remained (Figures 7B,D).

### 3.5 In *vitro* antibacterial effects

**FIGURE 8:**
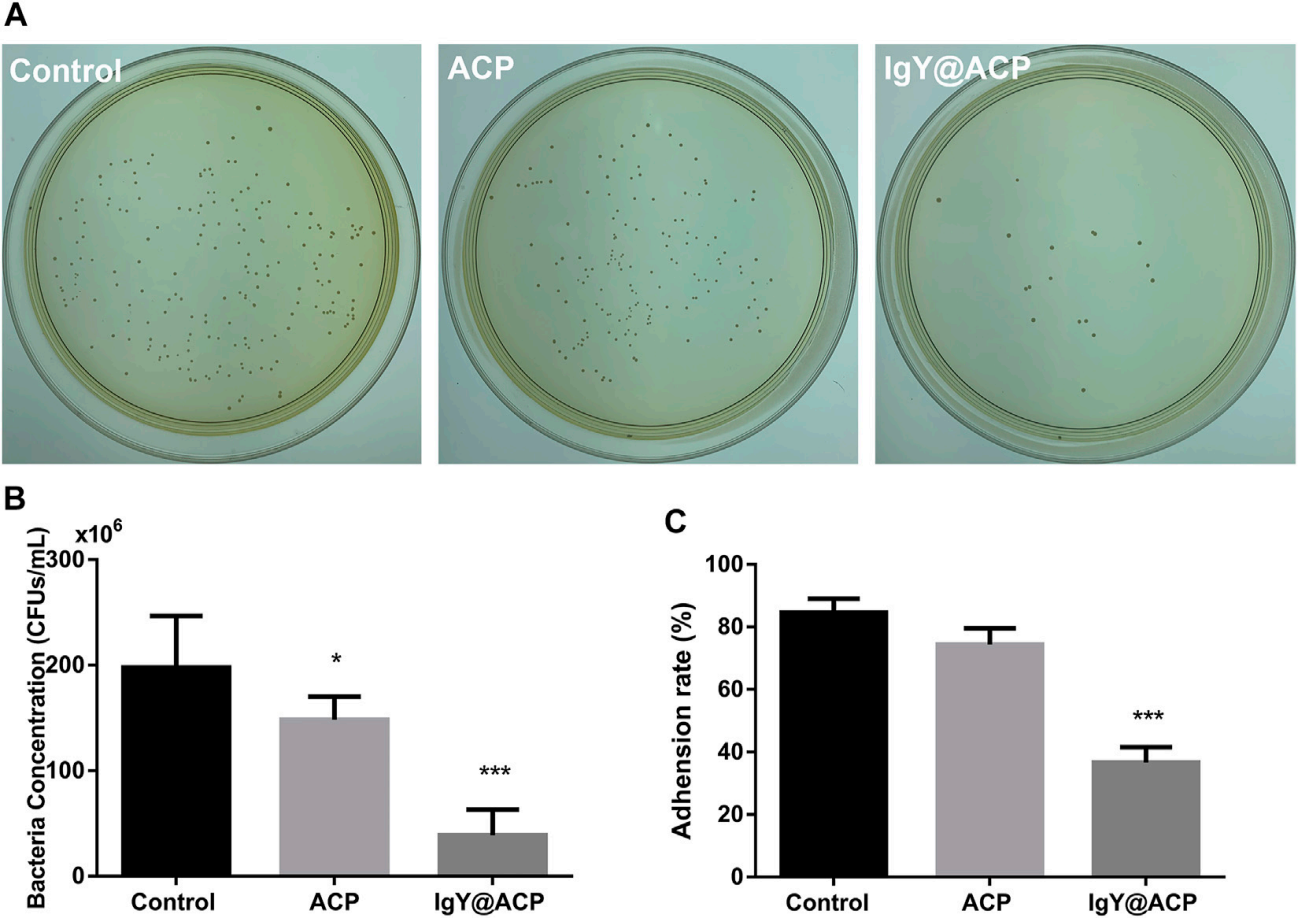
The antibacterial effects of IgY@ACP against planktonic *S. mutans* on CFU counting **(A,B)** and adhesion rate **(C)**. The significance level was set at *p < 0.05, **p < 0.01, and ***p < 0.001.

The antibacterial effects of IgY@ACP against planktonic *S. mutans in vitro* were evaluated by CFU counting and assessing the adhesion rate. As shown in Figures 8A,B, the CFU counting showed that the total number of viable bacterial colonies in the IgY@ACP group decreased significantly compared with that in the ACP and control groups (without any materials) (*p* < 0.001). The adhesion property of *S. mutans* was also evaluated after IgY@ACP treatment. The adhesion rate in the IgY@ACP group decreased significantly after incubation for 24 h, as shown in Figure 8C (*p* < 0.001).

**FIGURE 9:**
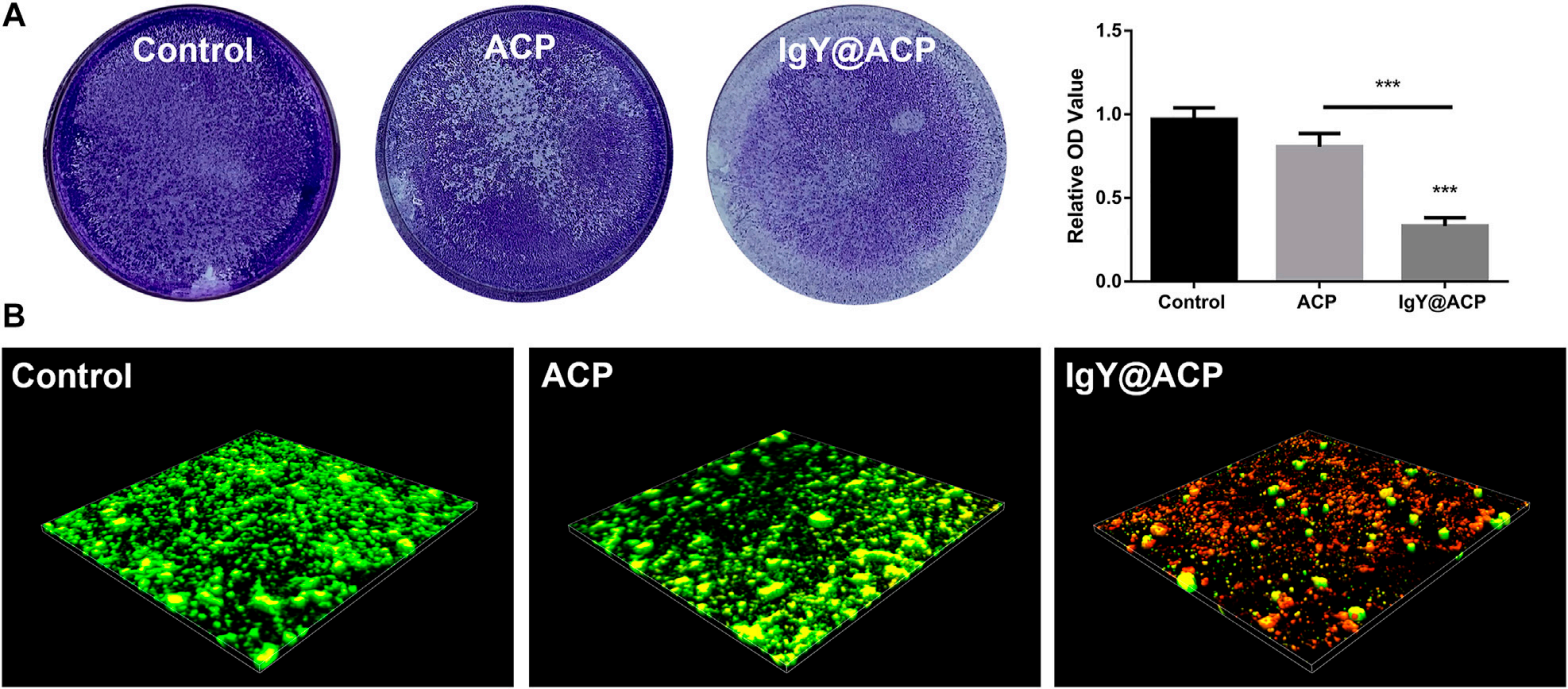
The biofilm formation of *S. mutans* after treatment with control, ACP, and IgY@ACP. Crystal violet staining with quantitative analysis **(A)** and live-/dead-stained 3D images **(B)**. Statistical significance was set at *p < 0.05, **p < 0.01, and ***p < 0.001.

The biofilm formation of *S. mutans* after treatment with IgY@ACP was evaluated, as shown in Figure 9. In Figure 9A, a dense biofilm structure can be observed with strong staining in the ACP and control groups. The IgY@ACP group presented a thin and loose structure with an obviously lighter staining color. The quantitative analysis showed that the OD value of the IgY@ACP group decreased significantly compared with that of the ACP and control groups (*p* < 0.001). Similar results were also found by CLSM after live/dead bacterial staining; green and red fluorescence imaging showed the live (green) and dead (red) bacterial distribution within the biofilms, respectively. As shown in Figure 9B, after treatment for 24 h, the biofilm structure of *S. mutans* in the IgY@ACP group displayed mostly red fluorescence, while that of *S. mutans* in the ACP and control groups displayed mostly green fluorescence.

## 4 Discussion

To treat DH by both occluding exposed dentinal tubules and inhibiting the main oral bacteria, a new desensitization material, IgY@ACP, was synthesized via biomimetic mineralization. Unlike the conventional strategy in which protein is simply adsorbed on prepared calcium phosphate, we loaded IgY in ACP using a biomimetic mineralization strategy. Briefly, IgY was mineralized with ACP in DMEM to form calcium phosphate products. Similar to simulated body fluid (SBF), DMEM has supersaturated Ca^2+^ and PO_4_^3-^ ions stabilized mainly by extra organic components, for example, amino acids and glucose (Tas, 2013; Tas, 2014; Fu et al., 2019). Upon further addition of Ca^2+^ ions, the stability is disturbed, and ACP forms with adsorbed IgY. TEM and SAED results showed that the products were amorphous as well as that the metastable feature favored the release of Ca^2+^ and PO_4_^3-^ ions and further promoted mineralization of dentin. The IgY@ACP particle size was approximately 60–80 nm, much smaller than the diameter of the dentinal tubule (4–8 μm), enabling it to enter the inner dentinal tubule to achieve deep occlusion. The FTIR results showed that the spectrum of IgY@ACP had a specific IgY peak (Chen et al., 2016), indicating that IgY molecules were successfully loaded into this calcium phosphate. Meanwhile, IgY@ACP exhibited a sustained release of protein for 48 h. At the early stage, the loaded IgY showed a relatively rapid release, which can be explained by the release of weakly adsorbed IgY molecules on the surface of the ACP nanostructure. Subsequently, IgY exerted a further continuous release, which played a key role in inhibiting *S. mutans* biofilm formation.

Biocompatibility is an essential consideration for materials used in biomedicine. In particular, the applied material for DH inevitably contacts mucosal tissue, as the defect related to DH is usually located at the cervical margin of the tooth (West et al., 2013). Therefore, HOK, a common cell in mucosal tissue, was selected to evaluate the cell biocompatibility of the prepared materials *in vitro.* In addition, considering that nanosized materials may influence healthy pulp tissue through dentinal tubules (Chiang et al., 2014), hDPSC viability was also investigated. The CCK-8 results showed that IgY@ACP had relative cell viability above 90% on HOK cells and hDPSCs. Specifically, amino acids and other organic components adsorbed on IgY@ACP from DMEM may improve the biocompatibility of calcium phosphate products (Schultheiss et al., 2013). In addition, IgY is an immune antibody produced by immunized hens and is commonly used in the form of mouthwash or toothpaste in the oral environment (Nguyen et al., 2011; Rahman et al., 2013). Therefore, the prepared IgY@ACP has reliable biosafety and application potential for DH treatment.

To evaluate the occluding performance of IgY@ACP in DH treatment, the NovaMin, which is used in clinics and toothpastes was chosen as the positive control. In our study, after treatment with IgY@ACP for 1 day, most of the exposed dentinal tubules were occluded, which was similar to that found in Iafisco M’s study, resulting from the consistent ion release (Iafisco et al., 2018). After treatment with IgY@ACP for 7 days, all dentinal tubules were completely occluded by a thicker material layer, more importantly, which extended into the tubules and the mineralized slices were interestingly found in the deep tubules up to a 16-μm depth. In addition, according to the XRD results, these dense minerals are similar to hydroxyapatite crystals, which are close to the natural dentin structure. The ACP phase could be separated and transformed into mature crystals to form bionic minerals in various solutions. Song J attempted to synthesize a nanogel containing ACP and then form crystals similar to dentin, thus achieving a surface-occluding thickness of 1–2 μm with a depth of 4–8 μm (Song et al., 2018). In addition, Berg Unosson synthesized ACP–magnesium particles and formed mineralized material in Tris-HCl buffer or saliva, which could even penetrate the tubules from the dentin surface up to 100 μm (Berg et al., 2020). We infer that a portion of IgY@ACP nanospheres penetrates into the dentinal tubules and then reacts with saliva, and Ca^2+^ and PO_4_^3-^ ions are repeatedly released and reprecipitated to form dense minerals on both the top surface and inner tubules, thereby occluding the dentinal tubules.

The oral acidic environment and acidophilic diet often impair the durable efficacy of desensitization treatment. Another challenge to the efficacy of desensitization treatment is daily abrasion. The acid resistance and abrasion resistance of the desensitization materials from IgY@ACP were evaluated. In our study, we used 6% (w/v) citric acid solution at pH = 1.5 to simulate the acidic conditions in the oral cavity, which is the main acidic component of fruit juices and beverages (Thanatvarakorn et al., 2013; Wang et al., 2015; Bastos-Bitencourt et al., 2021). The occluding materials on the dentin surface treated with IgY@ACP still existed, with little peeling and erosion traces after acid etching. Although acidic solutions may etch calcium phosphate components of the occluding materials on the dentin surface, the minerals in the dentinal tubules could continue to protect the tubules by further releasing Ca^2+^ and PO_4_^3-^ ions to promote remineralization (Huang et al., 2011). The mineralization of IgY@ACP shows a similar trend for the remineralization mechanism with acid resistance. Meanwhile, a soft-bristled toothbrush was used to imitate our daily abrasion. In the IgY@ACP group, the occluding materials on the dentin surface were worn away, but those in tubules remained due to their deep occlusion. Machado AC et al. evaluated the dentin permeability and tubule occlusion effects of several in-office desensitizing treatments containing calcium and phosphate and found that none of the products were able to resist acid and abrasive challenges (Machado et al., 2019). Researchers have been looking for improvements in the durable properties of desensitization materials. Our results indicated that loading IgY into ACP could improve the durability of calcium phosphate products.

Some researchers have attempted to load antibacterial materials, including antibiotics, metal ions, and other substances, into desensitization materials to improve their capacity to inhibit cariogenic bacteria (Fan et al., 2016; Huang et al., 2019; Jung et al., 2019; Wu et al., 2020). However, these improvements are limited to drug-resistant strains due to cytotoxicity, tooth discoloration, and other side effects. Antibacterial effects should be fully considered because caries often results in the worsening of DH teeth due to cariogenic bacteria. *S. mutans* is the main cariogenic bacterium in the early stages of caries. It forms biofilms that adhere to the tooth surface and cause tooth demineralization in the presence of fermentable carbohydrates (Chen et al., 2020). It is necessary to inhibit the growth of *S. mutans* while occluding the dentinal tubules. Our results showed that *S. mutans* was significantly decreased after treatment with IgY@ACP according to CFU counting changes, suggesting that IgY@ACP could effectively inhibit the growth of *S. mutans*. Furthermore, the number of tightly adhering bacteria decreased significantly in our study. It is believed that the biofilms formed by the bacteria adhering to each other on the tooth surface have stronger resistance to antibacterial drugs and play a vital role in protecting bacteria (Zhang et al., 2020). Biofilm formation following treatment with IgY@ACP was evaluated by crystal violet staining and CLSM, which showed a trend similar to IgY@ACP on *S. mutans*. Compared with the ACP and control groups, the IgY@ACP group showed lighter crystal violet staining and more dead bacterial cells. Therefore, IgY@ACP showed valuable antibacterial effects for IgY released from IgY@ACP. IgY could inhibit the glucosyltransferase (gtfs) activity of *S. mutans* and then inhibit their adhesion and aggregation (Bachtiar et al., 2016a; Wang et al., 2020). In addition, the continuous release of phosphate ions through calcium phosphate product degradation may play a non-negligible role, as these ions could relieve the effects of bacteria on teeth by neutralizing the oral acidic environment.

Until now, there have been few studies on DH *in vivo*, and there is no standard modeling metric. In our study, an animal model of DH was established *in vivo*. Pinto found that the depth between the pulp and enamel of mature rat incisors is approximately 1 mm (Pinto et al., 2009). The cavity preparation with a depth of 0.3 mm, which is equivalent to 1/3 of the above normal depth, will not damage the pulp tissue. In the control group, most of the dentinal tubules were exposed, indicating that the 0.3-mm depth could ensure the successful establishment of animal models simulating DH. In the IgY@ACP group, mineralized particles such as calcium phosphate were deposited on the dentin surface, which showed the occluding performance, while the antibacterial effect *in vivo* needed further experiments.

## 5 Conclusion

IgY@ACP could stably occlude dentinal tubules with acid challenge and effectively inhibit the growth and biofilm formation of *S. mutans*. These results suggest that IgY@ACP could be a promising desensitization material for DH therapy in the future. However, the limitation of IgY@ACP in achieving antibacterial and anti-caries effects should be evaluated over a long time *in vivo*. Meanwhile, there are different types of oral pathogenic bacteria in dental caries, and the improvement of biomaterials used for the inhibition of different caries-related pathologies will be further researched.

## Data Availability

The original contributions presented in the study are included in the article/Supplementary Material; further inquiries can be directed to the corresponding author.
